# Can Fundamental Movement Skill Mastery Be Increased via a Six Week Physical Activity Intervention to Have Positive Effects on Physical Activity and Physical Self-Perception?

**DOI:** 10.3390/sports4010010

**Published:** 2016-02-16

**Authors:** Elizabeth S. Bryant, Michael J. Duncan, Samantha L. Birch, Rob S. James

**Affiliations:** 1 London Sports Institute, Middlesex University, The Burroughs, London NW4 4BT, UK; 2 Research Centre for Applied Biological and Exercise Sciences, Coventry University, Priory Street, Covenrty CV1 5FB, UK; aa8396@coventry.ac.uk (M.J.D.); aa0052@coventry.ac.uk (S.L.B.); apx214@coventry.ac.uk (R.S.J.)

**Keywords:** fundamental movement skills, children, physical activity, obesity, intervention

## Abstract

Background: Previous research has suggested a positive relationship between fundamental movement skills (FMS) mastery and physical activity (PA) level. Research conducted on interventions to improve FMS mastery is equivocal and further research is needed. Methods: An intervention group of 82 children (35 boys and 47 girls) and a control group of 83 children (42 boys and 41 girls) were recruited from Years 4 and 5 (mean age ± SD = 8.3 ± 0.4 years) of two schools in Central England. The intervention included a combination of circuits and dancing to music. Pre and post intervention tests were conducted. Tests included: subjective assessment of eight FMS; objective measurement of two FMS; four day pedometer step count recording; height and mass for Body Mass Index (BMI); and the completion of Harter *et al.*’s (1982) self-perception questionnaire. Results: Following a two (pre to post) by two (intervention and control group) mixed-model ANOVA it was highlighted that the intervention group improved mastery in all eight FMS, and increased both daily steps and physical self-perception. Conclusions: It can be concluded that focussing one Physical Education (PE) lesson per week on the development of FMS has had a positive benefit on FMS, PA level and physical self-perception for the children in this study.

## 1. Introduction

The mastery of Fundamental Movement Skills (FMS) in children has been assessed in an attempt to identify any mechanisms underlying physical activity (PA) levels and weight status [1,2,3,4]. Research has recently identified that previous mastery in FMS can predict future PA levels in children [3,4,5]. These findings demonstrate the importance of FMS competency in childhood for maintenance of PA levels and in turn a healthy weight status. This has particular relevance for school Physical Education (PE) as the development of physical literacy and FMS is a key component of the National Curriculum for PE (NCPE) at key stages 1 and 2 in England [6].

Given this focus for school PE and the recent findings of Bryant *et al.*, (2014) [4], a key further step would be to determine whether it is possible to intervene and create an ecologically valid intervention to increase a child’s FMS competency to acquire the benefit of sustained PA participation. Primary school children in the United Kingdom currently have two hours of statutory PE per week [7]. This PE time needs to provide children with the skills and tools to be able to lead a physically active lifestyle [8]. An additional target for PE lessons proposed by the National Association for Sport and Physical Education [9] is that 50% of each lesson should be comprised of time in moderate-vigorous PA (MVPA) to contribute to the Department for Health’s “Start Active, Stay Active” daily target of at least 60 min of MVPA [10]. Consequently schools provide PE lessons to meet this target, often via team games [11,12]. However, this may be short-sighted, as engaging children in team games (to meet this target [9,13,14,15,16]) when they do not have mastery of FMS may be detrimental to future PA participation [17].

PE lessons that are less active but promote the teaching of skill-related fitness [16] may be more important in the long term to provide children with the skills to be physically active in later life [4]; which is the main goal in the NCPE [9]. According to Stodden *et al.*, (2008) [18] PA participation level and weight status are mediated by a combination of actual and perceived motor competence (PMC). Stodden *et al.*, (2008) [17] explains that when children reach middle to-late childhood (7–10 years old) their cognitive ability has developed to a stage where they can compare themselves to their peers [18]. Furthermore, children who obtain a higher PMC due to a higher actual motor competence (AMC) will perceive tasks as being easier and are more likely to engage in them. This engagement in PA will allow a child to practice and develop their AMC, sustain their PA level and maintain a healthy weight status [17]. Previous research has identified significant relationships between PMC, PA levels and weight status [19,20,21]. Although this significant relationship has been identified it needs to be distinguished as to whether PMC can be changed through an intervention to gain the benefit of an increased PA level. It is important to note that there are many factors that affect lifestyle behaviours, such as PA and weight status, and that motor development is just one such factor that contributes to this.

To date, no studies appear to have examined whether a short-term intervention, focusing on FMS during one out of two statutory school PE session, might result in greater benefit than engaging in two sessions of statutory PE per week. The current study sought to address this issue by: running an exploratory six week trial that replaced one out of two statutory PE lessons per week focused on the teaching of FMS; and determining the efficacy of such an intervention on PA, weight status and physical self-perception.

## 2. Method

### 2.1. Sample

Three primary schools in Central England were invited to take part in the study, of which two schools accepted. The schools were chosen from the same bracket of electoral wards (50%–59%) for deprivation and socio-economic status [22] as a means to ensure that extremes of affluence and deprivation did not confound the results of the study. When the schools were chosen, it was ensured that they were not currently, or had not previously been, part of any specialist PE programmes/interventions. Following informed consent from parents/guardians, 165 Years 4 and 5 children, mean (±SD) age of 8.3 ± 0.4 years, were recruited to take part in the study. Each school had two classes per year group. From the two classes per year group, one class was selected by a teacher to complete the intervention, whilst the other class was the control group. The intervention group consisted of 82 children (35 boys and 47 girls) and the control group consisted of 83 children (42 boys and 41 girls).

### 2.2. Study Design

The intervention group and control group were both tested to determine baseline values for FMS, daily steps, physical self-perception and weight status. The intervention group in each school underwent the 6 week intervention programme as detailed in Table 1. Only one PE lesson was replaced in an attempt to have little disturbance in the school’s curriculum, and to create a design that could be realistically repeated by class teachers in schools. The control group continued their normal physical education (PE) lessons. Immediately post completion of the intervention the control and intervention groups were both retested for FMS, daily steps, physical self-perception and weight status. Both schools involved in the intervention had two classes per year group; one class acted as the intervention group and one as the control group. Further details on all methods are given below.

**Table 1:** The Content of the Six Week Intervention (30 s rest between each activity).

Weeks 1–3	Aim	Content	Equipment (for 30 Children)
Warm up (5 min)	To steadily increase the children’s heart rate. Prepare the body for similar movements that they are about to undertake.	Dancing to a song (cha cha slide, DJ Casper). This song is popular for the age group completing the research and includes instructions that the children have to follow, for example, hopping, jumping and sliding.	CD player
Instruction time (8 min)	To identify key coaching points of each skill to the children.	Coaching points from the POC [23] were explained and demonstrated to the children.	N/A
Circuits 1. Running (3 min)	The use of the hurdles will encourage children to lift their knees higher to increase efficiency of the sprint [24].	Running over hurdles. Hurdles would start on the lowest level (10 cm) and increase in height each week (17.5 and 25 cm). Children were instructed to stay on the balls of their feet and drive their arms.	Five agility hurdles
2. Balance (3 min)	For children to control a balance board by engaging core muscles to improve balance.	Standing on balance boards. They were instructed to not let the board touch the floor. If they found this too easy then they were told to close their eyes. They were instructed to tense their stomach muscles.	Eight wobble boards
3. Throwing and catching (3 min)	For children to learn the correct technique to throw and catch a ball.	Children were in pairs standing five metres apart and practiced throwing and catching to each other with a tennis ball. They were given instructions for: the throw-start standing side on and step forward with the opposite leg; and the catch-meet the ball with your hands and bring it in to your body.	Eight tennis balls and 16 cones
4. Kicking (3 min)	For the children to learn the correct technique of the kick.	Children stood five metres from each other in pairs and kicked a ball back to each other. Children were instructed to take a step and swing back the leg, and keep their eye on the ball.	Eight small soft round playground footballs
Songs			
1. Jumping (Kriss Kross, Jump)	For children to learn the technique of the jump through a song that they will engage with.	Children were demonstrated the correct technique of a counter movement jump and were asked to complete this jump every time they heard the word jump in the song.	CD player
2. Hopping (One Direction, One way or another)	For children to learn the technique of hopping through a song that they will engage with.	Children were split into four teams. The children were demonstrated the correct technique of the hop with coaching points such as “stay on your toes”. Children then completed hopping relays in their teams.	CD player, cones
3. Gallop (PSY, Gangnam style)	For children to learn the technique of galloping through a song that they will engage with.	Children were demonstrated the correct technique of the gallop and given coaching points such as, “make sure your hips and shoulders stay straight and do not twist”. Children would gallop in a circle around the room and when the chorus came on they would all come to the middle of the hall and perform the “gangnam style” dance to the music.	CD player
Cool down-song (Harlum shake)	To progressively cool the body down with lighter exercise to decrease the children’s heart rate and to recap the movements learnt in the session.	Children had to pick a skill that they had performed in the session and mime it to the speed of the music. This was repeated three times with them choosing a different skill each time.	CD player
**Weeks 3–6**	**Aim**	**Content**	**Equipment (for 30 Children)**
Warm up (7 min)	To steadily increase the children’s heart rate. Prepare the body for similar movements that they are about to undertake.	Pirate ship game. Commands were shouted out to the children to which they had to perform a response. These were: Starboard: side gallop to the right hand side of the hall. Port side: hop to the left hand side of the hall. Climb the riggings: pretend to climb up a ladder getting their knees high and parallel to the ground. Scrub the deck: the children had to get onn the floor on their hands and knees pretending to wash the floor. Invasion: children had to pretend to throw bombs at other pirates. Spying: children had to pretend to throw bombs at invading pirates. Walk the plank: children had to stand on one leg as if they were on the end of a plank.	N/A
Instruction time (8 min)	To identify key coaching points of each skill to the children.	Coaching points from the POC^23^ were explained and demonstrated to the children.	N/A
Circuits 1. Hopping (3 min)	To be able to hop over all the hurdles without the use of the other leg.	Hurdles were set at the lowest level and children were asked to hop on one leg over each hurdle. Legs were then alternated between goes.	Five agility hurdles
2. Kicking (3 min)	To put the technique of the kick that the children have learnt into practise via competition.	Yellow stars were stuck on the wall and children had to kick and aim at the star. If they hit a star they scored one point. Children would compete against their partner.	Six cardboard stars and four small soft playground footballs
3. Throwing (3 min)	To put the technique of the throw that the children have learnt into practice via competition.	Multi coloured cut outs of hands were stuck onto a wall and children had to throw a tennis ball, aiming at the hands. If they hit a hand then they would score one point. Each child competed against their partner.	Six cardboard hands and four tennis balls
4. Jumping (3 min)	To complete the jump on a trampet to help increase height and encourage children to control the jump.	Children would do three consecutive jumps on a trampet, with the final jump landing on a mat in front.	Trampet
5. Balance (3 min)	To successfully walk along the benches without falling off.	Balance. Children would walk along three upside down gym benches with a cone on their head.	Three gymnasium benches
Relays (6 min)	To successfully complete the gallop and the run in a competitive situation.	Children remained in their five teams and completed running and galloping relay races. Children were demonstrated the correct technique and given coaching points.	10 cones
Cool down-game (7 min)	To play a more static game to decrease heart rate and to successfully catch the ball in a competitive situation.	Children were split into two teams. Within each team children had to stand in two lines with a partner in front of them. Children had to throw the ball back and forth, from person to person, and work it up the line so that everyone had to touch the ball. If someone dropped that ball then that team had to start again from the beginning. The first team to get the ball to the other end without dropping it were the winners. To begin with, children had to have their hands behind their backs before catching that ball. This was to prove that it is much harder to catch a ball if you don’t have your hands prepared to catch. It then would progress to having your hands waiting in a cupped position with a stable stance and as a consequence each team would complete the challenge quicker, therefore reinforcing the importance of being prepared.	Two tennis balls

#### 2.2.1. Instrumentation

##### Anthropometric Measurements

Anthropometric measurements were carried out individually in a sports hall prior to the performance of the fundamental movement skills (FMS). Children wore shorts and a t-shirt, but shoes were removed. Height (cm) and body mass (kg) were recorded to the nearest cm and 0.1 kg respectively using a stadiometer and electronic weighing scales (SECA Instruments Ltd., Hamburg, Germany), respectively. Body Mass Index (BMI) was calculated as Kg/m^2^. Children were classed as overweight if they were in the 85th centile and obese if in the 95th centile [25]. Using these criteria, 64% of children were classified as “normal weight” and 36% as “overweight/obese”.

#### 2.2.2. Fundamental Movement Skill Assessment

##### Subjective Measurement

FMS was assessed using the Process Orient Checklist (POC) [23]. Children were demonstrated each skill once with no coaching points. Children were asked to perform each skill three times. This checklist is comprised of eight different FMS; Sprint Run, Side Gallop, Hop, Kick, Catch, Overarm Throw, Vertical Jump and Static Balance. Each Skill is broken down into five or six components; depending on how many components the child demonstrated a percentage of mastery was calculated. The average percentage of mastery for the three trials was used for further analysis. In addition to the percentage of mastery being calculated, if a child demonstrated all components of the skill then they were rated as having “mastery”; if they demonstrated all but one of the components of the skill then “near mastery” and if more than one component were deemed to be missing then the participant was said to have no mastery of the skill [23].

Each FMS was video recorded (Sony video camera, Sony, Surrey, UK) and subsequently edited into single film clips of single skills on a computer using Quintic biomechanics analysis software (Quintic Consultancy Ltd., Sutton Coldfield, UK). Each video clip was given a unique identification code and did not indicate if the clip was from the intervention group or pre or post assessment. The clips were coded by a researcher who was not involved in analysis of the videos. This allowed researchers to be blinded to which group each participant was in and which stage of the research the participants were at to limit subjective and unconscious preferable scoring. The skills were also analysed using this software, enabling the videos to be slowed down, magnified and replayed. Prior to analysis inter- and intra-rater reliability analysis was performed for all the FMS between each researcher and the lead researcher. Inter-rater reliability was 91% and intra-rater reliability was 92%, demonstrating good reliability [20].

##### Objective Measurement

A 10 m sprint run was timed (s) using Smartspeed^®^ gates (Smartspeed, Fusion Sport, Coopers Plains, Queensland, Australia). Two laser gates were set up ten meters apart. The children were asked to sprint through the two gates. The height (cm) of the vertical jump was measured using a Myotest jump monitor (Myotest, Sion, Switzerland), as recommended by Bubanj *et al.*, (2010) [24]. The children had three attempts at each test and the best score was taken for analysis.

#### 2.2.3. Physical Activity Assessment

Habitual physical activity (PA) was measured using a sealed pedometer (Yamax Digi-Walker; SW 700, Yamax, TX, USA) worn over four days (two weekdays and two weekend days) [26]. Average daily steps were recorded for analysis. If children failed to collect data on any of the four days and/or values of <1000 steps or >40,000 steps per day were recorded on any day then all data for that child was excluded from the analysis in accordance with recommendations for the treatment of pedometer derived PA [27]. Children kept a diary of the step count that they completed each day. This diary was signed off by their parents to ensure that the legitimate pedometer data was recorded and used for analysis.

#### 2.2.4. Children’s Self-Perception of Skill Ability Assessment

A modified version of The Perceived Physical Competence Subscale for Children questionnaire (PPCSC) was used [18]. The questionnaire was reduced to 10 questions to represent popular activities which have been shown to have good discrimination between self-efficacy levels. The modifications made to the PPCSC replicated those made in other publications that have also utilized the PPCSC [28,29,30]. The questionnaire was completed in a classroom in the presence of the project coordinator and a class teacher.

### 2.3. Data Analysis

No children missed more than one session out of the six weeks, therefore data from all participants was retained and analysed.

Firstly, the Q-Q plot and the kurtosis and skewness values for each variable were assessed to identify whether each variable was normally distributed. The values for skewness and kurtosis were all between <1 and >−1 and therefore normally distributed [31], allowing the use of parametric testing.

Independent t test were conducted on baseline characteristics between the intervention group and the control group to ensure that they were comparable prior to intervention. The independent *t* tests highlighted no significant difference (*p* > 0.05) between the intervention (I) and control (C) group for gender (I: 46.6% boys and 53.4% girls and C: 50% boys and 50% girls), age (I: 8.35 ± 0.47 years and C: 8.34 ± 0.47 years), BMI (I: 18.01 ± 2.9 kg/m^2^ and C: 18.33 ± 3.3 kg/m^2^), average daily steps (I: 9682 ± 6109 steps and C: 10,036 ± 8527 steps) and overall motor competency percentage (I: 45.16% ± 15.02% and C: 47.34% ± 11.71%).

Children who had full mastery of a particular FMS at baseline, and therefore could no longer improve, were removed from the analysis of that FMS. *i.e.*, their data was only removed for the tasks that they showed full mastery for, not for all the FMS. This resulted in a number of children being removed from the FMS analysis as follows: Intervention boys: run 1; hop 0; gallop 9; catch 2; throw 2; kick 1; jump 0; balance 10. Intervention girls: run 0; hop 0; gallop 8; catch 8; throw 0; kick 0; jump 1; balance 15. Control boys: run 0; hop 0; gallop 11 catch 3; throw 3; kick 1; jump 0; balance 13. Control girls: run 0; hop 0; gallop 5; catch 6; throw 0; kick 0; jump 0; balance 14.

A two (pre to post) by two (intervention and control group) mixed model ANOVA was conducted to analyse any main effects and pre to post by group interactions for FMS mastery, daily steps, BMI, and physical self-perception scores. Levene’s test was used to determine whether variances were equal between each group. Where variances were unequal we have taken a more conservative approach and used *p* < 0.001 as the critical *p* value. Where significant interactions were highlighted, paired samples *t* test was conducted on the variable to identify if the intervention or control group significantly increased. Significance was set at *p* < 0.05.

## 3. Results

### 3.1. FMS Changes

#### 3.1.1. Subjective Measure

##### Boys

Following the two (pre/post) by two (intervention/control) mixed model ANOVA there was an overall main effect from pre to post for all eight fundamental movement skills (FMS) (All eight FMS *p* < 0.001 in each case).

A pre to post significant interaction between intervention and control group was highlighted for seven out of the eight FMS (Hop and gallop *p* < 0.05; catch, throw, kick, balance and jump *p* < 0.001).

**Figure 1:**
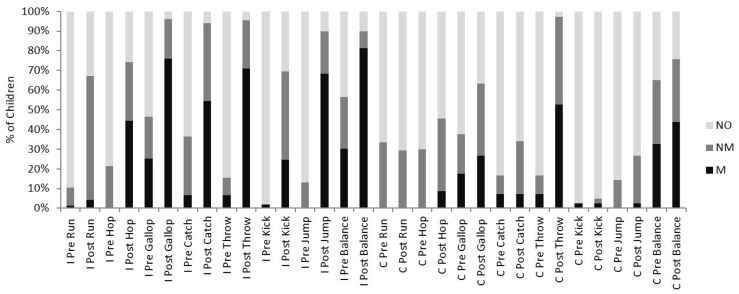
Percentage of Boys Classed as M (Mastery), NM (Near Mastery) and NO (No Mastery) for all eight fundamental movement skills (FMS) Pre and Immediately Post the Six Week Intervention (*N* = 35) and Control Group (*N* = 42). I = Intervention Group; C = Control Group.

Paired *t* tests highlighted that for the seven skills with a significant interaction there was a significant increase in skill mastery level between pre and post for the intervention group. (Intervention group, Hop, gallop, throw, kick, balance and jump *p* < 0.001; catch *p* < 0.5). For the same seven skills the paired samples *t* tests highlighted that five skills also significantly increased in mastery level for the control group (Hop, gallop, throw and jump *p* < 0.001). Although the control group also significantly improved from pre to post in five measures of FMS, the intervention group improved by a greater magnitude compared to the control group (Figure 1).

##### Girls

Following the two (pre/post) by two (intervention/control) mixed model ANOVA there was an overall main effect from pre to post for all eight FMS (All eight FMS *p* < 0.001).

**Figure 2:**
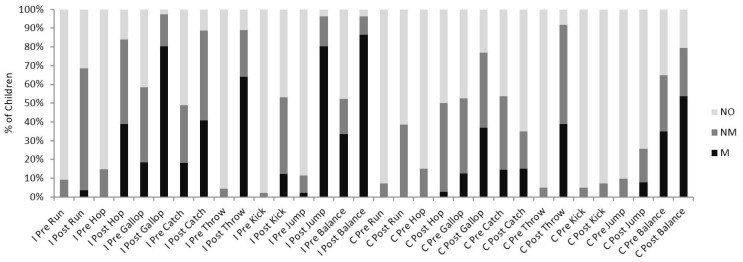
Percentage of Girls Classed as M (Mastery), NM (Near Mastery) and NO (No Mastery) for all eight fundamental movement skills (FMS) Pre and Immediately Post the Six Week Intervention (*N* = 47) and Control Group (*N* = 41). I = Intervention Group; C = Control Group.

A pre to post between intervention and control group significant interaction was highlighted for seven out of the eight FMS (Hop and gallop *p* < 0.05; catch, throw, kick balance and jump *p* < 0.001). Paired t tests highlighted that for the seven skills with a significant interaction there was a significant increase in skill mastery level between pre and post for the intervention group. (Intervention group, Hop, gallop, throw, kick, balance and jump *p* < 0.001; catch *p* < 0.05). For the same seven skills the paired samples *t* tests highlighted that five skills also significantly increased in mastery level in the control group (Hop, gallop, throw balance and jump *p* < 0.001) Although the control group also significantly improved from pre to post in five measures of FMS, the intervention group improved by a greater magnitude compared to the control group (Figure 2).

#### 3.1.2. Objective

##### Boys

There was a significant main effect for pre to post jump height for the intervention and control group (ANOVA *F*(1) = 10.3, *p* < 0.05). There was no significant interaction between the intervention and control group. There was no significant main effect for pre to post sprint speed for the intervention and control group (*f*(1) = 3.3, *p* > 0.05).

##### Girls

There was a significant main effect for pre to post jump height for the intervention and control group (ANOVA *F*(1) = 23.6, *p* < 0.05). There was no significant interaction between the intervention and control group for jump height. There was no significant main effect for pre to post sprint speed for the intervention and control group (*f*(1) = 1.58, *p* > 0.05). However, there was a significant interaction between the intervention and control group, pre to post assessments (*f*(1) = 4.46, *p* < 0.05). There was no significant difference from pre to post measurement of the sprint run for the intervention group (paired *t* test, *t*(46) 0.66, *p* > 0.05). However, there was a significant difference between pre to post measurement for the control group (*t*(40) −2.17, *p* < 0.05). The mean (±SD) for the sprint speed and jump height, pre and post intervention, for the intervention and the control groups are displayed in Table 2.

**Table 2:** Mean ± SD for objectively measured fundamental movement skills (FMS) pre and post intervention, for intervention (*N* = 35 boys and 47 girls) and control groups (*N* = 42 boys and 41 girls).

Gender	Intervention		Control	
Boys	Pre	Post	Pre	Post
Jump height (cm)	21.6 ± 4.9	25.3 ± 1.65	19.4 ± 6.4	21.3 ± 4.2 *
Sprint speed (sec)	2.09 ± 0.69	1.96 ± 0.86 *	2.18 ± 0.75	2.36 ± 0.45
Girls	Pre	Post	Pre	Post
Jump height (cm)	21.2 ± 4.0	23.2 ± 4.5 *	19.4 ± 6.4	21.3 ± 4.2 *
Sprint speed (sec)	2.41 ± 0.68	2.34 ± 0.85	2.18 ± 0.75	2.36 ± 0.45

Note: * Significantly different from pre intervention or control (*p* < 0.05).

#### 3.1.3. Body Mass Index

There was no significant change in body mass index (BMI) in the intervention or control group from pre to post measurements (ANOVA *p* > 0.05) for both boys and girls.

### 3.2. Physical Activity Changes

#### Boys

There was a significant main effect for the intervention and control group (ANOVA *F*(1) = 8.3, *p* < 0.05). However, there was no significant interaction between pre/post and the intervention and control group (*f*(1) = 0.71, *p* < 0.05).

#### Girls

**Figure 3:**
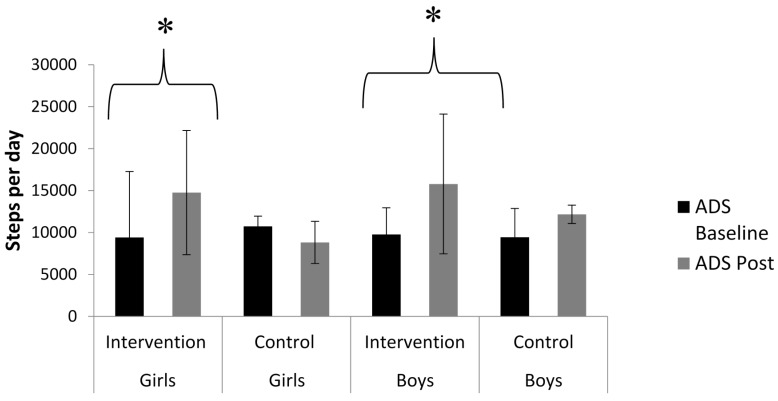
Mean (±SD) of Pedometer Step Counts for Girls and Boys For Intervention Group at Baseline, Post Six Weeks and for the Control Group at Baseline and Post Six Week Control. * Significant increase from baseline to post intervention (*p* < 0.05). (ADS: Average Daily Steps).

There was a significant main effect for the intervention and control group (ANOVA *F*(1) = 21, *p* < 0.001). There was also a significant interaction between the intervention and control group from pre to post intervention (*f*(1) = 13.7, *p* < 0.001). There was a significant increase in daily steps from pre to post (paired *t* test, *t*(20) −4.55, *p* < 0.001). However, there was no significant difference in daily steps from pre to post control group (*t*(18) 01.68), *p* > 0.05). Mean (±SD) average daily steps for pre and post intervention and control are displayed in Figure 3.

### 3.3. Physical Self-Perception

#### Boys

There was a significant main effect for the intervention and control group (*f*(1) = 4.75, *p* < 0.05) for physical self-perception scores. There was no significant interaction between the intervention and control group from pre to post measurement (*f*(1) = 0.016, *p* > 0.05).

#### Girls

There was a significant main effect for the intervention and control group (*f*(1) = 11.8, *p* < 0.001) for physical self-perception scores. There was no significant interaction between the intervention and control group from pre to post measurement (*f*(1) = 0.13, *p* > 0.05). The mean (±SD) PPSCS score for pre and post intervention and control are displayed in Table 3.

**Table 3:** The mean (±SD) for self-perception according to the Phyisical Percieved Competency Scale for Children (PPCSC) for pre and post intervention and control.

Gender	Intervention		Control	
Boys	Pre	Post	Pre	Post
PPCSC Score	28.94 ± 6	31.06 ± 4.6	27.18 ± 6.5	29.49 ± 6.1
Girls	Pre	Post	Pre	Post
PPCSC Score	26.14 ± 5.6	28.8 ± 6.5	27.24 ± 5.4	30.25 ± 5.47

## 4. Discussion

This current study has identified that the children who completed this intervention increased significantly more than the control group mastery of fundamental movement skills (FMS), pedometer step counts and physical self-perception.

The study is novel as it presents an ecologically valid FMS intervention (the materials and methods could realistically be repeated in a school by class teachers) which positively influences FMS, physical activity (PA) and physical self-perception. Stodden *et al.*, (2008) [17] identified that a combination of actual motor competence (AMC) and perceived motor competence (PMC) will influence PA and then weight status. To the author’s knowledge, no other study has identified whether all three of these variables will be influenced from an increase in AMC through an intervention. This study is also novel in that it sought to modify one school PE lesson per week rather than add additional PA sessions (such as work by Cliff *et al.*, (2007) [32]; Graf *et al.*, (2008) [33]) or to completely change the physical education (PE) curriculum [34]. Thus, not only is the intervention efficacious effective, it is also ecologically valid and feasible for primary schools to implement.

### 4.1. Overall Changes in FMS

The number of children who were classed as having mastery for all eight subjectively measured skills increased post intervention. This is consistent with the findings of Mitchell *et al.*, (2011) who reported a significant increase in the number of children who were classed as having mastery for all 12 FMS that were assessed after a minimum of a six week intervention. Mitchell *et al.*’s (2011) [35] intervention was conducted on children aged 5–12 years old which provided teachers with a FMS manual to use through PE lessons and fitness classes. At baseline, the children in Mitchell *et al.*’s (2011) [35] study had a higher amount of children classed as having mastery levels (21.4%–84.6%) compared to the children at baseline in this study (0%–25.3%). Post intervention, the percentage of children classed as having mastery of the skills in Mitchell *et al.*’s (2011) [35] study ranged from 49.8% to 98.3%, compared to 3.6%–81.2% in the present study. Although Mitchells *et al.*’s (2011) [35] study reports a larger amount of children as having mastery of the skills, the Test of Gross Motor Development 2 (TGMD-2) was used to assess the FMS. Although it is still a process orient based method, it only has 3–4 components for each skill and therefore it is much easier for children to gain mastery of the skills. This is similar for Hardy *et al.*, (2010) [36] who also used the TGMD-2 to assess FMS and reported that 20.5%–76% of children had mastery of the same FMS. In this current study the POC, which was used to assess FMS, has 5–6 components for each skill, therefore making it much harder to gain full mastery of them. Okely *et al.*, (2004) [2] also used the POC and also reported lower numbers of children classed as having mastery 0%–35%. Although two different scales have been used between this intervention and Mitchell *et al.*’s (2011) [35] intervention, the two skills with the lowest and highest percentage of children classed as having mastery, were also the lowest and highest skills post intervention. This finding suggests that if all skills are focused on throughout an intervention then they will develop at similar rates with the extremes still remaining the extremes.

Out of the two skills that were measured objectively, jump height significantly improved post intervention, but sprint speed did not. Research has identified that locomotor skills such as the sprint run are harder to master [37,38]. This is reflected in the subjective result of the sprint run, as it was the skill that the least amount of children could master. This suggests that the new/correct technique that the children have learnt is not yet autonomous and that the children still have to concentrate when performing the skill which inhibits their objective performance outcome of the sprint run. Further research is needed to identify how long it takes for each skill to become autonomous to influence the amount of time needed to be spent on each skill in PE lessons in primary school. When children learn a new skill for the first time, they will get to the autonomous stage of learning quicker compared to when having to re learn a new skill [39]. The objective measure of the sprint run was measured over 10 m, therefore assessing acceleration rather than sprint speed. The technique that the children would have learnt is appropriate for sprinting at speed, not the acceleration phase of the sprint. This might also help to explain why the children improved in the subjective measure, but not the objective measure. In future children should be allowed a flying start when having their technique of the sprint run assessed.

An, explanation as to why there are differing numbers for the amount of girls and boys gaining mastery of FMS could be gender bias in activities. Research has highlighted that boys favour activities that require locomotor skills such as running and girls favour creative activities such as dance and gymnastics [39]. This difference in gender and FMS performance is consistent with previous research [2,40,41,42]. The intervention group significantly gained mastery in all eight FMS and the control group significantly gained mastery in six out of the eight FMS. Initially, when the control group improved alongside the intervention group it suggested that the change was not down to the intervention. Although the control groups were in a different class they were in the same year group at the same school. Some of the children in the intervention group had friends in the control group and when they met them in the playground they taught them the skills they had learnt; this was reported to the lead researcher by the children who participated in the intervention sessions. This highlights the importance of using separate schools as control groups to control for this limitation. Although, the control groups did significantly improve, the significant interactions highlighted that the intervention group improved significantly more than the control group.

The findings from this study indicate that an intervention in one group of children has wider benefits by significantly increasing FMS mastery in another group of children. From the perspective of the children this is a positive consequence. As previously stated the number of children who are classed as having mastery of the FMS increases for both the intervention group and the control group. This is inconsistent with previous research that had control groups at different schools to the intervention groups [39]. However, the significant interactions highlighted that the intervention group improved significantly more than the control group. If the control group from this study was at a different school, improvements in the control group may have differed from these results.

### 4.2. Physical Activity Changes

Children who completed the intervention were associated with having higher daily steps compared to the children who did not complete the intervention. Daily steps significantly increased in post intervention compared to baseline for the intervention group. Van Beurden *et al.*, (2003) [43] also found a significant increase, by 7.8%, in time spent in MVPA post intervention. On the contrary, Cliff *et al.*, (2007) [32] reported an overall decrease of 18% of time spent in MVPA. It was explained that this decrease was due to a ceiling effect and that baseline measures were 20% higher than the average for children that age in the area [32]. There are limited studies that have reported PA data alongside an intervention for FMS, however when they have, it has been MVPA that has been reported. Conversely, Westerterp and Plasqui (2004) [44] highlighted that it is activities of low and high intensity that are the main contributors to a child’s PA level. Therefore, by measuring their habitual PA level, like in the current study, it includes all levels of intensity that will contribute to a child’s PA status. This allows for the results to provide a better indication of the overall effect that an increase in FMS will have on habitual PA, which has been highlighted to be important to health [41]. However, with the limitations that pedometers hold, as discussed in the limitations section below, this statement about the results must be taken with caution.

### 4.3. Changes in Self Perception Scores

Perceived Physical Competence Scales for Children (PPCSC) scores significantly increased from baseline to post intervention. Previous research has suggested that children who have a higher self-perception are more likely to have better FMS competency [19,20,21]. Ekeland *et al.*, (2004) [45] completed a systematic review on exercise/PA and self-esteem in children and concluded that exercise has positive short term effects on self-esteem. Whilst these studies have identified this relationship between the two variables, the cause and effect relationship has still remained unanswered. It could be that if children perceive themselves as being more competent, in turn perceiving tasks as being easier, then they spend more time participating in PA, resulting in an increase in ACM [46,47]. Or on the other hand, it could be argued that children who spend more time in PA allow themselves to develop these skills, thus increasing their PMC due to a higher AMC.

Due to no follow up assessment being conducted it is unknown whether the PPCSC scores revert back to their original levels. However, once children have mastery of a skill they are highly unlikely to lose it and according to Stodden *et al.*, (2008) [17] the age of the children in this study should be able to accurately perceive their AMC. Therefore, with the number of children who have gained mastery of the FMS it seems unlikely that the PPCSC score would revert back to its original score [17].

### 4.4. Limitations

Six weeks after the intervention had finished follow up assessments were conducted on the intervention group. Unfortunately it was not possible to conduct the follow up assessments on the control group; therefore this is why this data was not included in the analysis in this paper. Six weeks after the cessation of the intervention, children increased in mastery in six out of the eight skills (not the throw and kick) compared to immediately post intervention. For daily steps, children remained significantly higher compared to baseline, however they had significantly decreased from immediately post intervention, therefore highlighting the possibility that PA levels could revert back to original levels. Further research is needed with longer follow up assessments that are conducted on both the intervention and control group.

An additional key limitation of the present study was the use of the control group from the same year group and school as the intervention group. It was speculated that the reason for the increase in the percentage of children, in the control group, classed as having mastery was due to the children in the intervention group teaching the control group in the playground. Another limitation was the use of pedometers as it did not give the measurement of intensity of physical activity. Although the aim of the study was to assess habitual PA, if intensity had been measured it would have given information as to whether this intervention could have also contributed to the NICE PE target of 50% of their lesson at MVPA [10]. Furthermore, the intervention content involved diverse movements and types of activities that would not necessarily be identified by a step counter and therefore the intervention effect may have been underestimated. The generalisability of the results of this study is limited due to only two schools being used from the same area in central England. Therefore, it is unknown whether the same results would be seen in culturally different children.

## 5. Conclusions

It can be concluded that by focussing one PE lesson on the development of FMS (one hour per week for six weeks) it will have a positive benefit on PA level and physical self-perception. This study highlights a unique and efficacious way in which children could be taught the FMS. Teachers should take this information and use it to aid in the teaching of FMS. Using circuits as way of getting children to repeat the skill, but allowing them to move on quickly will enable children to stay engaged in the session whilst practising the skills. By interspersing the circuits with dancing to songs, it again keeps the children engaged and practising the techniques of skills. Although this intervention was in a six week block that was based on one school term in the UK, it can be adapted to suit any school structure by using the successful mechanisms elucidated in this intervention.

## References

[1] McKenzie T.L., Sallis J.F., Broyles S.L., Zive M.M., Nader P.R., Berry C.C., Brennan J.J. (2002). Childhood movement skills: Predictors of physical activity in anglo american and mexican american adolescents?. Res. Q. Exerc. Sport.

[2] Okely A.D., Booth M.L., Chey T. (2004). Relationships between body composition and fundamental movement skills among children and adolescents. Res. Q. Exerc. Sport.

[3] Barnett L.M., Morgan P.J., van Beurden E., Beard J.R. (2008). Perceived sports competence mediates the relationship between childhood motor skill proficiency and adolescent physical activity and fitness: A longitudinal assessment. Int. J. Behav. Nutr. Phys. Act..

[4] Bryant E.S., James R., Birch S., Duncan M. (2014). Does prior or current FMS predict future habitual physical activity (PA) levels and weight status?. J. Sports Sci..

[5] Okely A.D., Booth M.L., Patterson J.W. (2001). Relationship of physical activity to fundamental movement skills among adolescents. Med. Sci. Sports Exerc..

[6] (2013). Physical Education Programmes of Study: Key Stages 1 and 2. https://www.gov.uk/government/uploads/system/uploads/attachment_data/file/239040/PRIMARY_national_curriculum_-_Physical_education.pdf.

[7] Bardens J.B., Long R., Gillie C. (2012). School Report.

[8] Green K. (2008). Understanding Physical Education.

[9] Qualifications and Curriculum Authority (QCA) (2007). Physical Education: Programme of Study for Key Stage 3 and Attainment Target.

[10] Department of Helath (2011). Physical Activity Guidelines for Children and Young People. https://www.gov.uk/government/uploads/system/uploads/attachment_data/file/213739/dh_128144.

[11] Fairclough S.J. (2003). Physical activity levels during key stage 3 physical education 34. Br. J. Teach. Phys. Educ..

[12] Warburton P., Woods J. (1996). Observation of children’s physical activity levels during primary school physical education lessons. Eur. J. Phys. Educ..

[13] Hodges-Kulinna P., Martin J., Lai Q., Kliber A., Reed B. (2003). Student physical activity patterns: Grade, gender and activity influences. Br. J. Teach. Phys. Educ..

[14] McNamee G.D. (2005). The One Who Gathers Children: The work of vivian gussin paley and current debates about how we educate young children. J. Early Child. Teach. Educ..

[15] Trudeau F., Shephard R.J. (2005). Contribution of school programmes to physical activity levels and attitudes in children and adults. Sports Med..

[16] Stratton G., Fairclough S.J., Ridgers N.D., Smith A.L., Biddle S.J.H. (2008). Physical Activity Levels during the School Day. Youth Physical Activity and Sedentary Behaviour: Challenges and Solutions.

[17] Stodden D.F., Goodway J.D., Langendorfer S.J., Roberton M.A., Rudisill M.E., Garcia C. (2008). A developmental perspective on the role of motor skill competence in physical activity: An emergent relationship. Quest.

[18] Harter S. (1982). The perceived competence scale for children. Child Dev..

[19] Hands B. (2008). Changes in motor skill and fitness measures among children with high and low motor competence: A five-year longitudinal study. J. Sci. Med. Sport.

[20] Jones R.A., Okely A.D., Caputi P., Cliff D.P. (2010). Perceived and actual competence among overweight and non-overweight children. J. Sci. Med. Sport.

[21] Southall J.E., Okely A.D., Steele J.R. (2004). Actual and perceived physical competence in overweight and non-overweight children. Pediatr. Exerc. Sci..

[22] Coventry City Council (2004). Key Statistics. http://www.coventry.gov.uk/info/200088/information_and_statistics/749/key_statistics.

[23] New South Wales Department of Health (2003). Move It, Groove It—Physical Activity in Primary Schools Summary Report.

[24] Bubanj S., Stankovic R., Bubanj R., Bojiv I., Dindic B., Dimic A. (2010). Reliability of myotest test by a countermovement jump. Acta Kinesiol..

[25] Cole T.J., Freeman J.V., Preece M.A. (1990). Body mass index reference curves for the UK. Arch. Dis. Child..

[26] Trost S.G., Pate R.R., Freedson P.S., Sallis J.F., Taylor W.C. (2000). Using objective physical activity measures with youth: How many days of monitoring are needed?. Med. Sci. Sports Exerc..

[27] Rowe D.A., Mahar M.T., Raedeke T.D., Lore J. (2004). Measuring physical activity in children with pedometers: Reliability, reactivity, and replacement of missing data. Pediatr. Exerc. Sci..

[28] Horn T., Hasbrook C. (1987). Psychological Characteristics and the Criteria Children use for Self-Evaluation. J. Sport Psychol..

[29] Duncan T.E., Duncan S.C. (1991). A latent growth curve approach to investigating developmental dynamics and correlates of change in children’s perceptions of physical competence. Res. Q. Exerc. Sport.

[30] Kimiecik J., Horn T., Shurin C. (1996). Relationship among children's beliefs, perceptions of their parent’s beliefs, and their moderate to vigorous physical activity. Res. Q. Exerc. Sport.

[31] Kline R.B. (2005). Principles and Practice of Structural Equation Modelling.

[32] Cliff D.P., Wilson A., Okely A.D., Mickle K.J., Steele J.R. (2007). Feasibility of SHARK: A physical activity skill-development program for overweight and obese children. J. Sci. Med. Sport.

[33] Graf C., Koch B., Falkowski G., Jouck S., Christ H., Staudenmaier K., Tokarski W., Gerber A., Predel H., Dordel S. (2008). School-based prevention: Effects on obesity and physical performance after 4 years. J. Sports Sci..

[34] Jones R.A., Riethmuller A. (2011). Promoting fundamental movement skill development and physical activity in early childhood settings: A cluster randomized controlled trial. Pediatr. Exerc. Sci..

[35] Mitchell B., McLennan S., Latimer K., Graham D., Gilmore J., Rush E. (2011). Improvement of fundamental movement skills through support and mentorship of class room teachers. Obes. Res. Clin. Pract..

[36] Hardy L.L., King L., Espinel P., Okely A.D., Bauman A. (2011). Methods of the NSW schools physical activity and nutrition survey 2010 (SPANS 2010). J. Sci. Med. Sport.

[37] Schmidt R.A. (1975). A Schema theory of discrete motor skill learning. Psychol. Rev..

[38] Westendorp M., Houwen S., Hartman E., Visscher C. (2011). Are gross motor skills and sports participation related in children with intellectual disabilities?. Res. Dev. Disabil..

[39] Fitts P.M., Posner M.I. (1967). Human Performance.

[40] Blatchford P., Baines E., Pellegrini A. (2003). The social context of school playground games: Sex and ethnic differences, and changes over time after entry to junior school. Br. J. Dev. Psychol..

[41] Vandaele B., Cools W., de Decker S., de Martelaer K. (2011). Mastery of fundamental movement skills among 6-year-old flemish pre-school children. Eur. Phys. Educ. Rev..

[42] Bryant E.S., Duncan M.J., Birch S.L. (2014). Fundamental movement skills and weight status in british primary school children. Eur. J. Sports Sci..

[43] Beurden E.V., Barnett L.M., Zask A., Dietrich U.C., Brooks L.O., Beard J. (2003). Can we skill and activate children through primary school physical education lessons? move it groove it—A collaborative health promotion intervention. Prev. Med..

[44] Westerterp K.R., Plasqui G. (2004). Physical activity and human energy expenditure. Curr. Opin. Clin. Nutr. Metab. Care.

[45] Ekeland E., Heian F., Hagan K.B., Abbott J.M., Nordheim L. (2004). Exercise to improve self-esteem in children and young people. Cochrane Database Syst. Rev..

[46] Goodway J.D., Rudisill M.E. (1997). Perceived physical competence and actual motor competence of african american preschool children. Adapt. Phys. Act. Q..

[47] Weiss M.R., Amorose A.J. (2005). Children’s self-perception, in the physical domain: Between- and within-age variability in level, accuracy, and sources of perceived competence. J. Sport Exerc. Psychol..

